# COVID-VU – ENT-UK national survey of flexible nasendoscopy in the upper aerodigestive tract amidst the COVID-19 pandemic

**DOI:** 10.1186/s12913-021-07416-x

**Published:** 2022-05-09

**Authors:** Avgi Loizidou, Taranjit Singh Tatla, Ian Harvey, Miriayi Aibibula, Justin Roe, Neeraj Sethi, Anne G. M. Schilder

**Affiliations:** 1 Deptment of ENT-Head & Neck Surgery, London North West University Healthcare NHS Trust, London, Harrow HA1 3UJ UK; 2 School of Public Health, San Diego State University, San Diego, CA USA; 3 Ambu Limited, Incubator 2, Alconbury Weald Enterprise Campus, Alconbury Weald, Cambridgeshire, PE28 4XA UK; 4 Department of Speech, Voice and Swallowing, The Royal Marsden NHS Foundation Trust, London, UK; 5 National Centre for Airway Reconstruction, Department of Otolaryngology, Head and Neck Surgery, Imperial College Healthcare NHS Trust, London, UK; 6 Department of Surgery and Cancer, Imperial College, London, UK; 7 Dept of Otolaryngology-Head & Neck Surgery, Queens Medical Centre, Nottingham University Hospitals NHS Trust, Nottingham, NG7 2UH UK; 8 evidENT, Ear Institute, University College London, London, UK; 9 National Institute for Health Research University College London Biomedical Research Centre, London, UK

**Keywords:** Flexible nasendoscopy, COVID-19, Upper aerodigestive tract, Aerosol generating procedure

## Abstract

**Background:**

Flexible nasendoscopy (FNE) is an invaluable multi-disciplinary tool for upper aerodigestive tract (UADT) examination. During the COVID-19 pandemic concerns were raised that FNE had the potential of generating aerosols resulting in human cross-contamination when performed on SARS-COV2 carriers. In the UK, and other European countries, national guidelines were issued restricting FNE to essential cases. We surveyed ENT-UK members and Royal College of Speech and Language Therapists (RCSLT) members to determine the impact of the COVID-19 pandemic (first peak) on FNE practice in the UK.

**Methods:**

An observational internet-based survey constructed in accordance to the CHERRIES checklist and setup in SurveyMonkey of FNE practice amongst UK-based ENT surgeons and speech and language therapists in community clinics, the outpatient department, inpatient wards, ICU, emergency department and operating theatres (through the NHS and private sector) prior to, during and following the first COVID-19 wave in the UK.

**Results:**

314 responses collected (24% response rate), 82% from ENT clinicians, 17% from SLTs and 1% from other allied healthcare professionals. Overall, there has been a large reduction in the volume and indications for FNE during the first peak of the COVID-19 pandemic with limited recovery by mid-August 2020. Cancer and airway assessments were impacted less. A wide range of FNE protocols influenced by local factors are reported, varying in endoscope preference, Personal Protective Equipment (PPE) and sterilization methods. Where dedicated Aerosol Generating Procedure (AGP) rooms were unavailable, clinicians resorted to window opening and variable room “down-time” between patients. Endoscope preference reflected availability and user familiarity, ENT trainees favoring the use of single-use video endoscopes.

**Conclusion:**

Despite national guidance, local practice of FNE remains interrupted and highly variable in the UK. A collaborative inter-disciplinary approach is required to re-introduce FNE safely in volume across healthcare settings, re-establishing timely endoscopic diagnosis and pre-pandemic levels of patient care.

## Background

The COVID-19 pandemic altered clinical pathways and working practices in healthcare worldwide [1, 2]. Human-human transmission of the SARS-COV2 virus is by droplet spread and aerosolization [3]. At the start of the pandemic, flexible nasendoscopy (FNE) of the upper aerodigestive tract (UADT) was identified as a potential aerosol generating procedure (AGP), putting healthcare workers at risk when performing this in patients that may carry the SARS-COV2 virus. Studies confirm risk of transmission increases when patients cough or sneeze, which is common during nasendoscopy and endoscopic dysphagia assessments [4–6]. Patients initially free from SARS-COV2 may be at risk of its nosocomial transmission during the procedure of FNE, spread occurring through one or more routes: directly from healthcare workers operating the nasendoscope (i.e. asymptomatic carriers), from inadequate sterilization where re-usable nasendoscopes are used on consecutive patients, or from aerosolization when FNE is performed on neighboring patients in multi-bedded bays and open wards.

UADT endoscopy is undertaken in multiple clinical settings by multiple practitioners; it aids the diagnosis and management of upper airway obstruction, dysphagia and dysphonia, as well as tracheostomy tube trouble-shooting, weaning and decannulation. Speech and language therapists (SLTs) perform Endoscopic Evaluation of the Larynx (EEL) and Fibreoptic Endoscopic Evaluation of Swallowing (FEES). Multiple disciplines use UADT FNE to provide adult respiratory care [7–9]. Adjunctive endoscopy techniques permit tissue biopsies and aspirate samples to be taken, as well guide the safe insertion of percutaneous tracheostomies and appropriate siting of nasogastric tubes (NGT). Reducing UADT endoscopy practice to mitigate or avoid associated COVID-19 transmission risks may instead add to other non-COVID-19 patient health risks.

In March 2020, while there was much uncertainty on actual SARS-COV2 transmission risks during FNE, ENT-UK issued guidelines for enhanced PPE use and restricting nasendoscopy to essential cases, with concern of potential occupational health hazards of its members. Where perceived benefits of use outweighed risks, it was recommended FNE be performed in a well-ventilated room, wearing enhanced PPE (defined as Level 3 PPE consisting of: single use FFP3 mask, full length gown, face visor / goggles) and ideally using an endoscope with a camera screen [1, 2]. This guidance mirrored that from other national and pan-European ENT organisations, including CEORL-HNS, as reflected in the consensus statement of March 2020 [10–12]. Given the similarities for FNE guidance across Europe, UK observations for FNE activity and patterns of change during the pandemic may extrapolate to reflect a similar reality for other European states. In the field of gastroenterology, who also share concerns of aerosol generation and transmission of the SARS-COV2 virus when conducting endoscopy of the upper digestive tract; there is already published evidence of how these restrictions in guidelines issued has resulted in reduction in volume of endoscopy performed and subsequently delay in cancer diagnosis and treatment [13, 14]. Understanding the effects of these guidelines in the flied of otolaryngology may assist in the updating of UK and European guidelines, as well as help shape the development of future UK and Europe-wide health service delivery models.

### Aims and objectives

We aim to document how the first peak of COVID-19 and the issue of national guidance affected FNE practice in the acute and outpatient setting. In doing so, we look to consider obstacles and facilitators in clinical practice to re-establishment of multi-disciplinary FNE activity back to pre-pandemic levels.

## Methodology

An observational internet-based survey amongst UK-based ENT doctors and SLTs with EEL / FEES competencies performing FNE of the UADT in community clinics, outpatient department (OPD), inpatient wards, intensive care unit (ICU), emergency department (ED) and operating theatres.

### Outcomes measured

1. Indications and frequency of FNE of the UADT before, during and emerging from the COVID-19 first peak (16th March 2020 – 15th June 2020).
2. UADT endoscopy guidelines established before, during and emerging from the COVID-19 first peak.
3. Clinicians’ perspectives on the impact of COVID-19 on UADT endoscopy practice following the first pandemic peak.

### Data collection

A 21 question online survey was constructed in accordance to the CHERRIES checklist [15] and setup in SurveyMonkey [16], a well-established online data collection tool. Prior to release, the survey was quality checked through internal pilot by the co-authors to ensure that the questions were inclusive, clear and avoiding duplications. The survey was also subjected to external validation twice by leading academic SLTs serving on the RCSLT endoscopy Clinical Excellence Network (CEN) and by the ENT-UK survey guardians for quality assurance and to ensure the questions reflected the multidisciplinary audience it was aimed at. The final published survey reflects changes following both of these external reviews. Invitations to participate in the survey were sent via membership email lists to UK practicing ENT surgeons (courtesy of ENT-UK), and UK practicing SLTs with EEL competencies (courtesy of RCSLT CEN and SLT social media network); a total of 1305 potential questionnaire respondents. The survey was also publicized through respective newsletters, accessible through a web-based link, with appropriate data protections. Data was collected between 16th July and 15th August 2020 inclusive. Two email reminders were sent during this period. The Raosoft online sample size calculator was used to calculate the sample size required for descriptive studies as per the epidemiological standards described by Scott A. and Smith TM in 1969 [17–19]. To achieve a 5% margin error and 95% confidence interval based on a 50% response distribution 297 responses were required, aiming for an overall response rate greater than 20%.

### Data analysis

Results note total counts and response percentages. Response percentages were calculated based on the overall number of responses collected for individual questions, rounded to the nearest integer. FNE volume is defined as number (range) of FNE performed per month and FNE recovery rate denotes how quickly FNE volume returned to pre-pandemic levels in June 2020 after the COVID-19 first pandemic peak.

Full data on the study results are available from the corresponding author upon request.

## Results

### Survey respondents demographics

314 responses were collected, with a survey response rate of 24% (314/1305), the majority of responses provided by ENT consultants. Responder demographics including speciality, clinical practice and FNE experience are shown in Table 1.

**Table 1:** Survey responder demographics

**Responder specialty**	**Total responses (*n*** **= 314)**
ENT	256 (82%)
ENT consultants ≥10 years experience	106 (34%)
ENT consultants < 10 years experience	72 (23%)
ENT trainees (all ranks in training post)	41 (13%)
ENT ST6-8 (Registrar)	19 (6%)
ENT ST3-5 (Registrar)	18 (6%)
Basic surgical training (CT / SHO)	4 (1%)
Registrar grade (out of training post)	12 (4%)
Staff and Associate Specialists (SAS)	15 (5%)
Oral and Maxillofacial Surgery	2 (1%)
Oncology (Head and Neck)	28 (9%)
Speech and Language Therapists (SLTs)	54 (17%)
RCSLT level 1 EEL and or FEES	6 (2%)
RCSLT level 2 EEL and or FEES	18 (6%)
RCSLT level 3 EEL and or FEES	30 (9%)
Other (i.e. Nurse Practitioners)	9 (3%)
**Clinical Setting**	**Total responses (*n*** **= 314)**
University teaching hospital	180 (57%)
District general hospital with teaching commitment	116 (37%)
District general hospital without teaching commitment	18 (6%)
Community based triage centre	49 (16%)
Private hospital	6 (2%)
**Endoscopy experience**	**Total responses (*n*** **= 314)**
Performed 500+ nasendoscopies	234 (75%)
Performed 150-500 nasendoscopies	43 (14%)
Performed 50-150 nasendoscopies	20 (6%)
Performed < 50 nasendoscopies	16 (5%)

*RCSLT* Royal College of Speech and Language Therapy, *EEL* Endoscopic Evaluation of the Larynx, *FEES* Fibreoptic Endoscopic Evaluation of Swallowing

### Changes in flexible nasendoscopy (FNE) use around the first COVID-19 peak

The first peak of COVID-19 resulted in a significant reduction in volume of FNE across all clinical settings for all clinical indications, except where airway and cancer concerns. After clinical activity resumed, recovery was very modest in the outpatient setting, slower in acute care, and slower still in the community (see Table 2).

**Table 2:** FNE volume by clinical setting and indication before, during and after the COVID-19 first peak

**Monthly FNE levels**	**Before COVID-19 (*n*** **= 270)**	**During COVID-19 (*n*** **= 269)**	**After COVID-19 (*n*** **= 266)**
< 5	13 (5%)	133 (54%)	72 (27%)
5-10	34 (13%)	67 (25%)	73 (27%)
11-50	102 (38%)	55 (20%)	101 (38%)
> 50	121 (45%)	3 (1%)	20 (8%)
**FNE according to clinical setting**	**Before COVID-19**	**During COVID-19**	**After COVID-19**
Community clinic (*n* = 62)	61 (98%)	6 (10%)	15 (24%)
Outpatient department (*n* = 249)	245 (98%)	170 (68%)	209 (84%)
Inpatient ward (*n* = 200)	197 (99%)	121 (62%)	150 (75%)
Emergency departments (*n* = 131)	128 (97%)	87 (66%)	96 (73%)
Operating theatres (*n* = 83)	76 (92%)	47 (57%)	56 (67%)
ICU (*n* = 156)	150 (96%)	88 (56%)	93 (60%)
**FNE according to clinical indication**	**Before COVID-19**	**During COVID-19**	**After COVID-19**
Epistaxis (*n* = 154)	152 (99%)	48 (31%)	81 (53%)
Airway assessment (*n* = 224)	222 (99%)	178 (79%)	196 (88%)
Foreign body ingestion / inhalation (*n* = 162)	162 (100%)	99 (61%)	115 (71%)
Cancer assessment (*n* = 205)	201 (98%)	157 (77%)	175 (85%)
Swallow assessment (*n* = 225)	221 (98%)	78 (35%)	157 (70%)
Voice assessment (*n* = 240)	237 (99%)	86 (36%)	160 (67%)
Aid passage of NG tube (*n* = 123)	123 (100%)	38 (31%)	52 (43%)
Aid insertion of tracheostomy tube (*n* = 80)	78 (98%)	36 (45%)	44 (55%)
Aid tracheostomy care / decision-making (*n* = 161)	154 (96%)	80 (50%)	112 (70%)

**Fig. 1:**
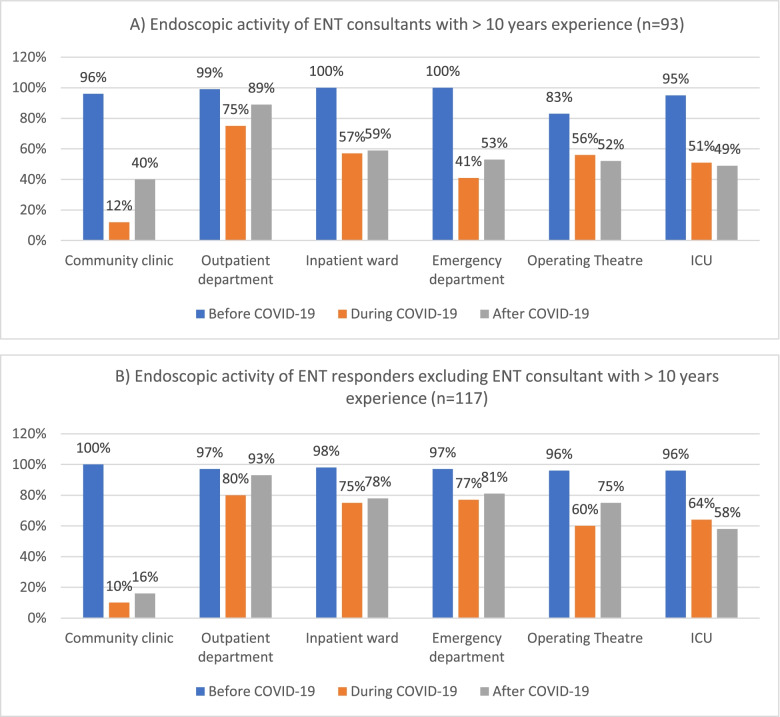
Endoscopy activity before, during and after the COVID-19 first peak of A) ENT consultant with > 10 years experience and B) all other ENT responders

ENT consultants with ≥10 years experience resumed endoscopic activity faster in the outpatient setting than the acute care environment. The opposite was observed by consultants with < 10 years experience, junior doctors (ENT registrars, ENT Core and Higher Surgical Trainees [CT / HSTs] and Staff and Associate Specialist [SAS] doctors) (see Fig. 1).

SLT responders demonstrated higher recovery rates in the acute setting, with FNE volume for airway assessment, tracheostomy management and swallow assessment showing greater resumption compared to voice assessment (see Table 3).

**Table 3:** FNE activity according to clinical setting before, during and after the first COVID-19 peak

**FNE activity according to clinical setting**	**Before COVID-19**	**During COVID-19**	**After COVID-19**
Community clinic (*n* = 3)	3 (100%)	0 (0%)	0 (0%)
Outpatient department (*n* = 29)	29 (100%)	2 (7%)	11 (38%)
Inpatient ward (*n* = 41)	41 (100%)	12 (30%)	34 (83%)
ICU (*n* = 31)	30 (97%)	13 (42%)	23 (74%)
**FNE activity according to clinical indication**	**Before COVID-19**	**During COVID-19**	**After COVID-19**
Airway assessment (*n* = 14)	14 (100%)	7 (50%)	13 (93%)
Cancer assessment (*n* = 1)	1 (100%)	0 (0%)	0 (0%)
Swallow assessment (*n* = 47)	47 (100%)	16 (34%)	37 (79%)
Voice assessment (*n* = 29)	29 (100%)	8 (28%)	15 (52%)
Aid passage of NG tube (*n* = 2)	2 (100%)	0 (0%)	1 (50%)
Aid tracheostomy care / decision-making (*n* = 29)	27 (93%)	13 (45%)	23 (79%)

### Changes in local clinical guidelines around the first COVID-19 peak

49%(*n* = 132) responders state their department had FNE Standard Operating Procedures (SOPs) prior to the pandemic, 66%(*n* = 154) report local SOPs established during the first peak and 12%(*n* = 29) state new SOPs were established while emerging out of the first peak. 85%(*n* = 40) of SLT departments had specific SOPs/guidelines for FNE before the pandemic, compared to only 41%(*n* = 90) of ENT departments. These figures may be underestimated as 25% (*n* = 14) of ENT trainees and 12% (*n* = 11) of ENT Consultants > 10 years experience report being unsure if departmental SOPs for FNE were present prior to the pandemic.

Of local SOPs (where existent), 48%(*n* = 105) didn’t specify which type of nasendoscope to use during the COVID-19 peak, 32%(*n* = 70) allowed both single and re-usable nasendoscopes and only 2%(*n* = 5) specified using single-use nasendoscopes.

SOPs were largely consistent in support for enhanced PPE (91%,*n* = 153), hand hygiene (79%,*n* = 134), the use of video monitor nasendoscopes (73%,*n* = 123) and a dedicated AGP room (72%,*n* = 121) where available. Other directives were highly variable, including how AGP rooms were to be used and the room “down-time” between examinations (see Table 4).

**Table 4:** Features present in Standard Operating Protocols (SOPs) of ENT departments across the country in response to the first COVID-19 wave

**Standard operating protocols (*n*** **= 168)**	**Responses**
Practicing hand hygiene	134 (80%)
Patient wearing a standard surgical mask during the procedure	78 (46%)
Enhanced PPE (FFP3 mask, full length gown, face visor or goggles)	153 (91%)
Enhanced decontamination of re-usable nasendoscope	49 (29%)
Introduction of single-use nasendoscope	47 (28%)
Video nasendoscope with-screen monitor	123 (73%)
Dedicated room for nasendoscope decontamination	65 (39%)
Dedicated AGP room	121 (72%)
Other changes	19 (11%)
**AGP room specifications (*n*** **= 142)**	**Responses**
Designated negative pressure room	14 (10%)
Negative pressure room with portable HEPA filtration unit	6 (4%)
Open windows	56 (39%)
Dedicated room – no negative pressure or open window	44 (31%)
Room left for 20′ after cleaned	42 (30%)
Room left for 20-60′ after cleaned	46 (32%)
Room left for 60-120′ after cleaned	17 (12%)
Room left for less than 20′ after cleaned (no coughing or sneezing during procedure)	33 (23%)
Other changes	20 (14%)

### Choice of endoscopes around the first COVID-19 peak

A wide range of nasendoscopes are used in clinical practice: re-usable eye-piece fibreoptic nasendoscopes (portable, direct eye viewing nasendoscope) (67%,*n* = 181), re-usable video nasendoscopes (64%,*n* = 171), and single-use video nasendoscopes connecting to a re-usable digital screen monitor (portable screen allows shared viewing, with image/video playback and recording) (18%,*n* = 49). Multiple factors influence the users choice of nasendoscope, the main being nasendoscope familiarity (55%,*n* = 113), its ease of use (50%,*n* = 102), option for video/image capture (49%,*n* = 101) and nasendoscope availability (48%,*n* = 98).

96%(*n* = 45) of SLTs reported use of re-usable video nasendoscopes compared to only 11%(*n* = 5) using re-usable eye-piece fibreoptic nasendoscopes and 13% (*n* = 5) using single-use video nasendoscopes. The trend is reversed for ENT responders with 80%(*n* = 174) using re-usable eye-piece fibreoptic nasendoscopes, 57%(*n* = 125) using re-usable video nasendoscopes and 20%(*n* = 44) using single-use video nasendoscopes.

### FNE preferences emerging from the first COVID-19 peak

Following the first COVID-19 peak, ENT users reported a shift in nasendoscope preference with 23%(*n* = 40) selecting single-use video nasendoscopes, 36%(*n* = 63) reusable nasendoscopes and 29%(*n* = 62) using a combination of both depending on clinical circumstances. Of ENT responders excluding consultants, 34%(*n* = 16) expressed preference for reusable nasendoscopes, 36%(*n* = 17) for both re-usable eye-piece (fibreoptic) and single use video nasendoscopes, according to clinical need and 9%(*n* = 5) indicated no preference. In contrast only 20%(*n* = 23) of ENT consultants reported a preference for single-use video nasendoscopes, while 55%(*n* = 39) prefer re-usable video screen nasendoscopes and 26% use either according to clinical indication.

### Profiling endoscope decontamination practices around the first COVID-19 peak

Prior to the pandemic, decontamination was mainly carried out with Tristel (chlorine dioxide) wipes (74%,*n* = 185) and central sterilization (50%,*n* = 126). Little change was noted in decontamination methods deployed in response to the COVID-19 first peak.

Concerningly, a proportion of responders reported central sterilization was rarely or never used (41%, *n* = 16 of SLT respondents, 23%, *n* = 42 of ENT respondents). Both during and emerging from the first peak, SLT responders note an overall reduction in central sterilization service use.

During the COVID-19 first peak, 20%(*n* = 45) of departmental scope disinfections were carried out in the same room as the FNE assessment, 46%(*n* = 104) were carried out in a separate disinfection room, and only 33%(*n* = 74) were carried out in a central sterilization unit. ENT trainees and SLT colleagues were more likely to clean scopes in the same room that the FNE took place (22%, *n* = 11 and 29%, *n* = 12 respectively), compared to ENT Consultants with > 10 years of experience (15%, *n* = 12).

### Endoscope traceability and cross-contamination around the first COVID-19 peak

Overall, respondents note that endoscopy logbooks and traceability stickers were used across all clinical settings (ICU 88%(*n* = 106), ED 90%(*n* = 86), inpatient wards 94%(*n* = 130) and the OPD 96%(*n* = 64)). When asked for historical report of endoscope cross-contamination, 86% (*n* = 213) responded no cases, 10% (*n* = 25) were unaware of any such incidences and only 4% (*n* = 11) expressed knowledge or experience. However, ENT trainees reported up to 21%(*n* = 7) of ICUs and 18%(*n* = 6) of EDs did not have traceability stickers and logbooks locally.

### Obstacles in re-establishing endoscopy activity levels after the first COVID-19 peak

67%(*n* = 135) quoted local restrictions as the main obstacle for restoring nasendoscopy activity to pre-pandemic levels. These restrictions included an ongoing requirement for a (variable) time interval between FNE examinations, to permit room cleaning and ventilation, and so reduce potential AGP risk (see Table 5).

**Table 5:** Obstacles Preventing Re-establishing Endoscopy to Pre-pandemic Levels (*n* = 203)

Limited supply of PPE	29% (*n* = 59)
Inadequate tariff payments to cover additional cost of providing enhanced PPE for endoscopist and assistants (for suspected or unknown COVID-19)	23% (*n* = 46)
Inadequate number of single use scopes	25% (*n* = 51)
Damaged reusable scopes	9% (*n* = 19)
Lack of video monitors for single-use scopes	27% (*n* = 54)
Reusable scopes not cleaned in time causing delay in service delivery	18% (*n* = 37)
Handling and cleaning safety issues for reusable scopes	14% (*n* = 29)
Lack of familiarity and training for single use scopes	9% (*n* = 18)
Limited access to reusable scope camera stack	30% (*n* = 61)
Not enough reusable scopes	13% (*n* = 27)
Local restrictions imposed on time interval between scopes and to allow for room cleaning and ventilation (due to potential AGP risk)	67% (*n* = 135)
Adherence to local guidelines for use of alternative radiological investigations instead of nasendoscopy (due to potential AGP risk)	11% (*n* = 23)
Other ENT	10% (*n* = 21)

## Discussion

### Study strengths and limitations

This was a comprehensive and widely distributed questionnaire completed shortly after the first COVID-19 peak in the UK, collecting a large volume of responses from experienced endoscopy users; it can therefore be considered a good proxy for experience in the UK. However, the reported findings reflect the experience and work pattern of the responders during the first COVID-19 peak, responder bias being a major limitation. The majority of responses were from ENT consultants, the results depicting more accurately the effects of the first COVID-19 peak in the OPD setting where they provided most service. Additional sub-group analysis is needed to explore the experience of ENT trainees and SLTs in the acute setting (inpatient wards, ED, ICU etc). Furthermore, the survey did not attempt to identify individual centres and so we are unable to assess how clinical practice may have changed according to regional variation in COVID-19 pressures or how the difference in resources of the individual centres restricted the changes that were possible. While we expect similar results for other European countries, individual national studies would be needed to verify this, similarly with consideration for responder bias.

### Impact of the first Covid-19 peak on nasendoscopy activity

The value of UADT nasendoscopy lies in the anatomical and functional information it provides, particularly its suitability for detecting early-stage curable head and neck mucosal cancers. Experience with COVID-19 has highlighted, in addition to the importance of OPD and inpatient capacity, the importance of guidelines / SOPs, essential facilities and resources, equipment and consumables to prevent cross-contamination and so reduce infection transmission risks. The absence of such infrastructure, equipment and safeguards has inevitably reduced nasendoscopy volume.

Many ENT departments during the first COVID-19 peak turned to radiology to offset the diagnostic gap created by reduced nasendoscopy activity. Unfortunately, in the context of early stage UADT cancer, radiological tests provide limited information in the absence of correlative endoscopy, often leading to distraction and over-investigation of other coincidental radiological findings. This has implications for adding to healthcare costs, radiology diagnostic capacity and further delays to the patient’s diagnostic and treatment pathway.

Evidence is emerging of delay in head and neck cancer diagnosis during the first COVID-19 peak, an increase in later stage disease cancer presentations, and missed early cancer re-occurrences post-treatment [19]. A recent systematic review and meta-analysis reveals even 4 weeks of delay in cancer treatment is associated with an increase in mortality [20]. CovidSurg recently reported that while head and neck surgery can be safely delivered during the Covid-19 pandemic [21], treatment pathways have altered with a reduction of patients referred for surgery (more being directed to chemo-radiotherapy options), as the requirement for inpatient care during the COVID-19 pandemic bed crisis was a deterrent. Thus, reduction in nasendoscopy activity during the COVID-19 first peak has inevitably impacted the care pathway of head and neck cancer patients in multiple ways.

### Guidance

Early during the pandemic, guidelines issued by ENT-UK noted that nasendoscopy had the potential to become an AGP if the patient sneezes or coughs during the examination [1, 2]. The guidance specifies methods to reduce risk of aerosols, including patients wearing a surgical mask during the examination, FNE conducted in a separate AGP room, and performed with a camera screen for viewing, so permitting the wearing of enhanced PPE including a face and eye-shields. ENT-UK guidance clearly states that a prolonged gap between clinical encounters is only necessary if there has been aerosol generation during nasendoscopy. However, our results suggest that in the “real-world” experience of local NHS services acting in an environment of pervasive uncertainty, protocols and practice continues to assume that much, if not all, of nasendoscopy is an AGP, so inevitably inhibiting its prompt and ready use.

In January 2021, Public Health England published the recommendations of the Independent High Risk AGP Panel on medical procedures carried out in the UK which do not currently meet the current World Health Organization (WHO) definition for high risk AGPs [22]. Due to lack of sufficient evidence for increased risk of infection in nasendoscopy, the independent AGP Panel was unable to call nasendoscopy a high-risk AGP. The validity of this conclusion is debated as it was derived from weak evidence which emerged from SARS-CoV1 and MERS pandemics. Moreover, currently there is limited research on the risk of cross-contamination through direct contact of the nasendoscope with the UADT of SARS-CoV2 positive patients.

Unfortunately, despite the guidance issued, variability in endoscopy practice and risk mitigation continues within the ENT community as well as across other endoscopic services. Specialties including upper GI endoscopy and thoracic bronchoscopy, have been able to continue service delivery by introducing additional safety measures such as performing endoscopy in ventilated endoscopy suites and screening patients with COVID-19 PCR swabs prior to the procedure [23]. If the same effect is to be observed in nasendoscopy, efforts need to be made to improve communication of continuously changing guidance to local departments and encourage the uptake of risk mitigation measures endorsed by sister specialties.

Recognising the limitation of the current evidence base for potential or actual risk in AGPs, the National Institute for Health Research (NIHR) has recently established a multidisciplinary Task and Finish group to help guide on AGP research priority areas. This is a significant and key outcome which is very much welcomed by all.

Early in the first wave of the COVID-19 pandemic, various UK professional bodies, including ENT-UK, RCSLT and British Laryngological Association (BLA), each issued their own individual society guidance aiming to protect and safeguard their members from COVID-19 hazards [1, 2]. Unfortunately, inconsistencies in these recommendations caused some reported confusion and unnecessary tension in the clinical setting. Hereon, we recommend these organisations cooperate to develop consensus working groups along with other key national stakeholders (including infectious diseases and gastroenterology), to evaluate evidence-base and identify gaps in research, releasing joint multi-disciplinary team guidance.

### FNE risk in the acute care setting during COVID-19 pandemic

ENT trainees and SLTs, both working primarily in the acute care (non-OPD) setting during the first COVID-19 wave, indicate that the higher risk of exposure to COVID-19 acted as an ongoing barrier to the re-introduction of nasendoscopy. Contrasting to the OPD setting, the volume of FNE was unpredictable, by nature clinically urgent, and often performed on COVID-19 positive (or suspected) patients in a variety of acute care clinical spaces. These localities are even less likely to have suitably equipped AGP rooms than the ENT OPD, with enhanced PPE also not always available.

Traditionally, FNE performed at the patient’s bedside has been reliant upon eye-piece viewing through re-use fiberoptic nasendoscopes. While easy to carry, their use during COVID-19 exposes the user to potentially greater risk of SARS-COV2 transmission. Eye protection (face visor or goggles) is integral to enhanced PPE and this provides a barrier to direct eye-piece nasendoscopy viewing. Moreover, greater physical proximity between endoscope user and patient is needed so that the eye-piece can be viewed, increasing the user's risk of exposure to aerosols. Unlike the video nasendoscopy options, it does not permit the recording or sharing of digital image data, unless the eye-piece is connected to a separate camera and screen (these usually are only available in operating theatres and the outpatient setting). If a second clinical opinion is required, or closer scrutiny needed of endoscopic detail, the FNE examination needs to be repeated with further compounded risks for additional AGP. Thus, ENT trainees and SLT reliant upon fibreoptic eye-piece viewing for FNE, may have restricted its use to limited and essential cases, a sharp contrast to their unrestricted pre-pandemic practice. In ENT units such as those of the authors in London and Nottingham, the ready availability and virtues of digital single-use video nasendoscopes have permitted safer, more innovative practices to evolve for local ENT service delivery, trainee supervision and remote senior support amidst the COVID-19 pandemic.

During the first COVID-19 peak, decontamination methods were reported inconsistent, and the cases of nasendoscopes being sent for central sterilization was reduced. ENT trainees reported a 4-5 times greater contamination risk in the acute care setting compared to OPD, highlighting this as a potentially under-reported and under-recognised issue, raising added safety concerns for inpatients and junior staff safety. In the absence of evidence that nasendoscopy carries zero / negligible risk of SARS-COV2 transmission, nasendoscope traceability and decontamination arrangements become paramount. Single-use nasendoscopes interfaced with viewing screens, providing point of care video-recording and data sharing capabilities were preferred by 34% of ENT trainees (*n* = 16). Benefits included ease of nasendoscope disposal, avoiding risks inherent to endoscope cleaning, as well as the ability to remotely share endoscope images and video, so allowing increased levels of senior support for acute inpatient care. In the absence of a consistent, consensus, multi-disciplinary standard / SOP for FNE decontamination, the single-use video nasendoscope emerges as the default and safest FNE option for the acute care setting amidst the COVID-19 pandemic.

### Ongoing FNE risks as COVID-19 becomes endemic

When the SARS-COV2 virus was initially detected in Wuhan China, epidemiological effects akin to the winter flu were envisaged [22–27]. Unfortunately, the SARS-COV2 virus has demonstrated itself to be neither seasonal, nor confined by country or geographical borders [28]. New mutations of more virulent and deadly strains have been detected across the globe [29]. Throughout Europe, timings for COVID-19 peaks and troughs have been demonstrated to be discordant between countries, epidemiologists warning it is likely to become an endemic disease in such context [30–32]. While vaccination programs begin to show evidence of reduction in viral spread and patient mortality from the SARS-COV2 virus, we remain eager to see these benefits replicated in clinical practice during the annual winter months [33]. Otolaryngology, by nature of the profession, is inevitably disproportionately affected by the risk of SARS-COV2 virus transmission [34].

FNE of the UADT has become established as an invaluable and irreplaceable component of our clinical practice on multiple fronts, not least due to allowing the direct examination of the mucosa to screen, aiding the diagnosis of earlier stage cancer / pre-cancer / recurrent cancer, which is not possible with standard radiological investigations. In the context of acute care pathways too, its role is clearly established for assessing airway patency and reducing airway compromise risks associated with head and neck infection. It is unrealistic to consider that we can provide high quality care without it in the diagnostic armamentarium. Current guidelines therefore require multidisciplinary consensus for revision across the clinical environment (OPD and inpatient localities), adjustment and standardization to ensure safety for all patients and all healthcare staff. The expectation is that COVID-19 strains will continue to evolve and case numbers remain high intermittently throughout the year. Furthermore, guidance should ideally future-proof for the emergence of other potential transmissible infections, similar air-borne pathogens, as well as for possible iatrogenic cross-transmission of pathogens on the surfaces of re-usable endoscopes and surgical instruments. Concerns continue to be raised for prions especially evading endoscope sterilization, risking future outbreaks for conditions like variant Creutzfeldt-Jakob disease (a.k.a. mad cow’s disease) [35].

## Conclusion

The COVID-19 pandemic has unexpectedly stress tested health care services around the globe. While ENT-UK was prescient in offering guidance during this uncertain time, diagnostic activity has fallen dramatically in all settings. It has been slow to recover after the first peak, and this remains the situation following the second COVID-19 peak in the UK. Concerns for spread of the virus and cross-contamination have left clinicians cautious when undertaking FNE, prioritising more acute and critical cases and in continued low volumes.

This research demonstrates how provision patterns of UADT endoscopy have varied widely, often reflecting local guidelines where these were present, devised in many cases in a reactive manner on limited evidence-base. Whilst we are only able to comment on UK FNE observations, we expect similar results and patterns to be observed across Europe, highlighting shared concerns and risks across geographical boundaries. FNE has become an indispensable part of ENT clinical examination, helping inform and guide patient management at point of care. To resurrect previous FNE practice and resume established patient care, we need to rapidly re-introduce FNE back into our clinical practice with safety and agreement. Establishment of harmonised, consensus guidelines for the multidisciplinary team, inclusive of all stakeholder groups is urgently required. Evidence-base should be incorporated where present, or gaps clearly highlighted where absent, permitting commissioning and delivery of suitably designed and delivered multi-disciplinary research.

Whilst this may have been the first modern day global pandemic of an airborne virus, it is unlikely to be the last. Learning from the outcomes and impact of this pandemic provides an opportunity to evolve our clinical practice for the better: to become more suitable for multi-disciplinary integrated care delivery and ensure safety for all patients and healthcare staff alike. Revised guidance should be future-proofed and sustainable to withstand continuing COVID-19 peaks or other novel air-borne pathogen pandemics, as well as include risk reduction measures for other non-air-borne transmissible human diseases.

## Data Availability

All data is available upon request from the authors. Summary of the raw results submitted as supplementary information along with the main manuscript.

## Abbreviations

AGP: Aerosol generating procedure
BLA: British Laryngological Association
CEN: Clinical Excellence Network
CEORL-HNS: Confederation of European Otorhinolaryngology - Head and Neck Surgery
COVID-19: Coronavirus disease
CT: Core trainee doctors
ED: Emergency department
EEL: Endoscopic Evaluation of the Larynx
ENT: Ear nose and throat
FEES: Fibreoptic Endoscopic Evaluation of Swallowing
FFP3: Filtering Face Piece level 3 protection
FNE: Flexible nasendoscopy
NGT: Nasogastric tubes
HST: Higher Specialty trainees doctors
ICU: Intensive care unit
MERS: *Middle* East respiratory syndrome
NHS: National Health Service
*NIHR*: National Institute for Health Research
OPD: Outpatient department
PCR: *Polymerase chain reaction*
PPE: Personal protective equipment
RCSLT: Royal College of Speech and Language Therapists
SARS-COV2: *Severe acute respiratory syndrome coronavirus 2*
SARS-COV1: *Severe acute respiratory syndrome coronavirus 1*
SAS: Staff and Associate Specialist doctors
SLT: Speech and language Therapists
SOPs: Standard Operating Procedures
UADT: Upper aerodigestive tract
UK: United Kingdom
WHO: World Health Organization
